# Aqua­{2-morpholino-*N*-[1-(2-pyrid­yl)ethyl­idene]ethanamine-κ^3^ *N*,*N*′,*N*′′}bis­(thio­cyanato-κ*N*)cobalt(II)

**DOI:** 10.1107/S160053681100136X

**Published:** 2011-01-15

**Authors:** Nura Suleiman Gwaram, Nurul Azimah Ikmal Hisham, Hamid Khaledi, Hapipah Mohd Ali

**Affiliations:** a Department of Chemistry, University of Malaya, 50603 Kuala Lumpur, Malaysia

## Abstract

In the title complex, [Co(NCS)_2_(C_13_H_19_N_3_O)(H_2_O)], the Co^II^ ion is six-coordinated by the *N*,*N*′,*N*′′-tridentate Schiff base, the N atoms of two thio­cyanate ligands and one water mol­ecule in a distorted octa­hedral geometry. Intra­molecular C—H⋯N and C—H⋯O hydrogen bonds occur. In the crystal, inter­molecular O—H⋯O, O—H⋯S, C—H⋯S and S⋯S [3.5546 (18) Å] inter­actions result in an infinite three-dimensional network.

## Related literature

For the crystal structure of the analogous Ni^II^ complex, see: Suleiman Gwaram *et al.* (2011 ▶). For a similar Co(II) complex, see: Sun *et al.* (2007 ▶).
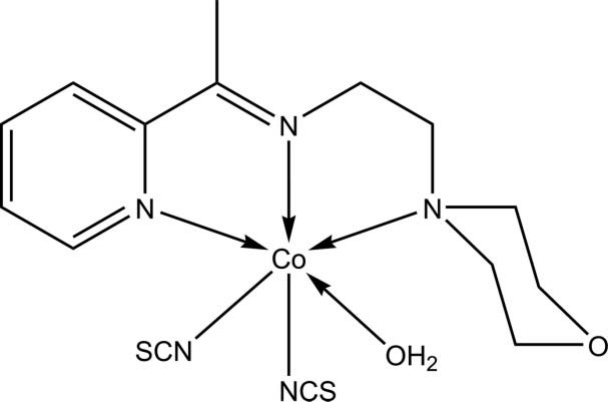


## Experimental

### 

#### Crystal data

[Co(NCS)_2_(C_13_H_19_N_3_O)(H_2_O)]
*M* *_r_* = 426.42
Monoclinic, 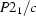

*a* = 7.1554 (3) Å
*b* = 22.187 (1) Å
*c* = 12.1297 (5) Å
β = 91.115 (3)°
*V* = 1925.31 (14) Å^3^
*Z* = 4
Mo *K*α radiation
μ = 1.13 mm^−1^
*T* = 100 K
0.18 × 0.10 × 0.08 mm

#### Data collection

Bruker APEXII CCD diffractometer
Absorption correction: multi-scan (*SADABS*; Sheldrick, 1996 ▶) *T*_min_ = 0.823, *T*_max_ = 0.915
13017 measured reflections
3401 independent reflections
2662 reflections with *I* > 2σ(*I*)
*R*_int_ = 0.062

#### Refinement

*R*[*F* ^2^ > 2σ(*F* ^2^)] = 0.055
*wR*(*F* ^2^) = 0.137
*S* = 1.08
3401 reflections
233 parameters
3 restraints
H atoms treated by a mixture of independent and constrained refinement
Δρ_max_ = 0.79 e Å^−3^
Δρ_min_ = −0.54 e Å^−3^

### 

Data collection: *APEX2* (Bruker, 2007 ▶); cell refinement: *SAINT* (Bruker, 2007 ▶); data reduction: *SAINT*; program(s) used to solve structure: *SHELXS97* (Sheldrick, 2008 ▶); program(s) used to refine structure: *SHELXL97* (Sheldrick, 2008 ▶); molecular graphics: *X-SEED* (Barbour, 2001 ▶); software used to prepare material for publication: *SHELXL97* and *publCIF* (Westrip, 2010 ▶).

## Supplementary Material

Crystal structure: contains datablocks I, global. DOI: 10.1107/S160053681100136X/pv2377sup1.cif

Structure factors: contains datablocks I. DOI: 10.1107/S160053681100136X/pv2377Isup2.hkl

Additional supplementary materials:  crystallographic information; 3D view; checkCIF report

## Figures and Tables

**Table 1:** Hydrogen-bond geometry (Å, °)

*D*—H⋯*A*	*D*—H	H⋯*A*	*D*⋯*A*	*D*—H⋯*A*
O2—H2*A*⋯O1^i^	0.83 (3)	1.88 (3)	2.701 (4)	172 (5)
O2—H2*B*⋯S1^ii^	0.83 (3)	2.36 (3)	3.161 (3)	162 (5)
C11—H11*A*⋯O2	0.99	2.40	3.121 (6)	130
C12—H12*B*⋯N4	0.99	2.62	3.511 (7)	150
C2—H2⋯S1^i^	0.95	2.85	3.774 (6)	165

Symmetry codes: (i) 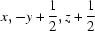; (ii) 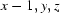.

**Fig. 1.**
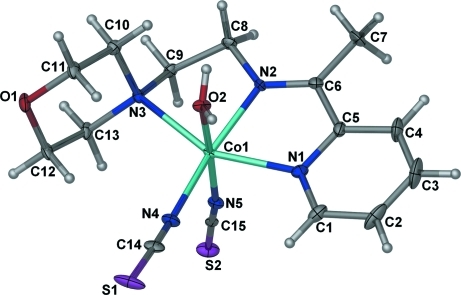
Thermal ellipsoid plot of the title compound at the 30% probability level. Hydrogen atoms are drawn as spheres of arbitrary radii.

## supplementary crystallographic information

### Comment

The crystal structure of the title Co^II^ complex is isostructural with the previously reported Ni^II^ complex (Suleiman Gwaram *et al.*, 2011). The Schiff base, prepared *in situ*, acts as an *N,N',N''*-tridentate ligand towards the Co^II^ ion to form two five-membered chelate rings with the metal atom. Two *cis*-located isothiocyanate and one water molecule complete a distorted octahedral geometry around the Co(II) center. A similar arrangement was observed in a related Co^II^ complex (Sun *et al.*, 2007). The molecular structure contains intramolecular C—H···N and C—H···O hydrogen bonds. In the crystal, the adjacent molecules are connected *via* O—H···O, O—H···S, C—H···S hydrogen bonds into infinite layers parallel to the *ac* plane. The layers are further linked into a three-dimensional polymeric structure through an S···S interaction [3.5546 (18) Å] between S1 and S2 of the symmetry related molecule at –*x+1*, *y* - 1/2, -*z* + 3/2.

### Experimental

A mixture of 2-acetylpyridine (0.20 g, 1.65 mmol) and 4-(2-aminoethyl)morpholine (0.21 g, 1.65 mmol) in ethanol (20 ml) was refluxed for 2 hr followed by addition of a solution of cobalt(II) acetate tetrahydrate (0.41 g, 1.65 mmol) and sodium thiocyanete (0.134 g, 1.65 mmol) in a minimum amount of water. The resulting solution was refluxed for 30 min, then left at room temperature. The crystals of the title complex were obtained in a few days.

### Refinement

The C-bound H atoms were placed at calculated positions (C–H 0.95–0.99 Å) and were treated as riding on their parent C atoms. The O-bound H atoms were located in a difference Fourier map, and refined with a distance restraint of O–H 0.84±0.02. For all H atoms, *U*iso(H) was set to 1.2–1.5 *U*eq(carrier atom). An additional rigid-bond type restraint (DELU in *SHELXL97*) was placed on the displacement parameters of S2 and C15.

### Crystal data

**Table:**

[Co(NCS)_2_(C_13_H_19_N_3_O)(H_2_O)]	*F*(000) = 884
*M**_r_* = 426.42	*D*_x_ = 1.471 Mg m^−^^3^
Monoclinic, *P*2_1_/*c*	Mo *K*α radiation, λ = 0.71073 Å
Hall symbol: -P 2ybc	Cell parameters from 2128 reflections
*a* = 7.1554 (3) Å	θ = 2.5–23.4°
*b* = 22.187 (1) Å	µ = 1.13 mm^−^^1^
*c* = 12.1297 (5) Å	*T* = 100 K
β = 91.115 (3)°	Rod, red
*V* = 1925.31 (14) Å^3^	0.18 × 0.10 × 0.08 mm
*Z* = 4	

### Data collection

**Table:**

Bruker APEXII CCD diffractometer	3401 independent reflections
Radiation source: fine-focus sealed tube	2662 reflections with *I* > 2σ(*I*)
graphite	*R*_int_ = 0.062
φ and ω scans	θ_max_ = 25.0°, θ_min_ = 1.8°
Absorption correction: multi-scan (*SADABS*; Sheldrick, 1996)	*h* = −8→8
*T*_min_ = 0.823, *T*_max_ = 0.915	*k* = −26→26
13017 measured reflections	*l* = −14→14

### Refinement

**Table:**

Refinement on *F*^2^	Primary atom site location: structure-invariant direct methods
Least-squares matrix: full	Secondary atom site location: difference Fourier map
*R*[*F*^2^ > 2σ(*F*^2^)] = 0.055	Hydrogen site location: inferred from neighbouring sites
*wR*(*F*^2^) = 0.137	H atoms treated by a mixture of independent and constrained refinement
*S* = 1.08	*w* = 1/[σ^2^(*F*_o_^2^) + (0.0626*P*)^2^ + 2.7605*P*] where *P* = (*F*_o_^2^ + 2*F*_c_^2^)/3
3401 reflections	(Δ/σ)_max_ < 0.001
233 parameters	Δρ_max_ = 0.79 e Å^−^^3^
3 restraints	Δρ_min_ = −0.54 e Å^−^^3^

### Special details

**Table:**

Geometry. All e.s.d.'s (except the e.s.d. in the dihedral angle between two l.s. planes) are estimated using the full covariance matrix. The cell e.s.d.'s are taken into account individually in the estimation of e.s.d.'s in distances, angles and torsion angles; correlations between e.s.d.'s in cell parameters are only used when they are defined by crystal symmetry. An approximate (isotropic) treatment of cell e.s.d.'s is used for estimating e.s.d.'s involving l.s. planes.
Refinement. Refinement of *F*^2^ against ALL reflections. The weighted *R*-factor *wR* and goodness of fit *S* are based on *F*^2^, conventional *R*-factors *R* are based on *F*, with *F* set to zero for negative *F*^2^. The threshold expression of *F*^2^ > σ(*F*^2^) is used only for calculating *R*-factors(gt) *etc*. and is not relevant to the choice of reflections for refinement. *R*-factors based on *F*^2^ are statistically about twice as large as those based on *F*, and *R*- factors based on ALL data will be even larger.

### Fractional atomic coordinates and isotropic or equivalent isotropic displacement parameters (Å^2^)

**Table:**

	*x*	*y*	*z*	*U*_iso_*/*U*_eq_	
Co1	0.14436 (7)	0.37659 (2)	0.77043 (5)	0.01682 (19)	
S1	0.62351 (16)	0.22817 (6)	0.78138 (16)	0.0511 (5)	
S2	0.40771 (17)	0.56976 (6)	0.67930 (12)	0.0357 (3)	
O1	0.1265 (5)	0.29112 (14)	0.4482 (3)	0.0315 (8)	
O2	0.0220 (4)	0.28962 (14)	0.7921 (3)	0.0248 (7)	
H2A	0.063 (7)	0.2640 (18)	0.836 (3)	0.030*	
H2B	−0.091 (4)	0.282 (2)	0.788 (4)	0.030*	
N1	0.1427 (5)	0.38088 (16)	0.9493 (3)	0.0240 (8)	
N2	−0.1063 (4)	0.41983 (16)	0.8045 (3)	0.0195 (8)	
N3	0.0131 (5)	0.38505 (15)	0.5984 (3)	0.0180 (8)	
N4	0.3878 (5)	0.32766 (17)	0.7616 (3)	0.0273 (9)	
N5	0.2780 (5)	0.45788 (16)	0.7493 (3)	0.0227 (8)	
C1	0.2665 (7)	0.3574 (2)	1.0199 (4)	0.0345 (12)	
H1	0.3780	0.3403	0.9924	0.041*	
C2	0.2382 (10)	0.3571 (3)	1.1326 (5)	0.0522 (17)	
H2	0.3300	0.3407	1.1817	0.063*	
C3	0.0743 (11)	0.3811 (3)	1.1727 (5)	0.064 (2)	
H3	0.0506	0.3807	1.2495	0.076*	
C4	−0.0533 (9)	0.4055 (3)	1.0995 (4)	0.0449 (14)	
H4	−0.1662	0.4224	1.1255	0.054*	
C5	−0.0178 (6)	0.4054 (2)	0.9889 (4)	0.0271 (11)	
C6	−0.1499 (6)	0.4296 (2)	0.9038 (4)	0.0269 (11)	
C7	−0.3223 (7)	0.4624 (3)	0.9403 (5)	0.0456 (15)	
H7A	−0.3986	0.4735	0.8755	0.068*	
H7B	−0.2855	0.4989	0.9808	0.068*	
H7C	−0.3949	0.4361	0.9883	0.068*	
C8	−0.2204 (6)	0.4400 (2)	0.7098 (4)	0.0246 (10)	
H8A	−0.2712	0.4807	0.7241	0.030*	
H8B	−0.3265	0.4120	0.6974	0.030*	
C9	−0.0980 (6)	0.4413 (2)	0.6091 (4)	0.0223 (10)	
H9A	−0.1778	0.4466	0.5423	0.027*	
H9B	−0.0121	0.4762	0.6145	0.027*	
C10	−0.1111 (6)	0.33506 (19)	0.5629 (4)	0.0232 (10)	
H10A	−0.1853	0.3478	0.4973	0.028*	
H10B	−0.1992	0.3259	0.6226	0.028*	
C11	−0.0029 (7)	0.2791 (2)	0.5357 (4)	0.0279 (11)	
H11A	0.0669	0.2650	0.6021	0.033*	
H11B	−0.0905	0.2468	0.5124	0.033*	
C12	0.2525 (7)	0.3375 (2)	0.4824 (4)	0.0296 (11)	
H12A	0.3422	0.3452	0.4229	0.036*	
H12B	0.3243	0.3238	0.5483	0.036*	
C13	0.1525 (7)	0.3950 (2)	0.5091 (4)	0.0255 (10)	
H13A	0.0872	0.4103	0.4420	0.031*	
H13B	0.2446	0.4258	0.5334	0.031*	
C14	0.4866 (6)	0.2874 (2)	0.7706 (4)	0.0297 (11)	
C15	0.3316 (5)	0.5040 (2)	0.7189 (4)	0.0192 (9)	

### Atomic displacement parameters (Å^2^)

**Table:**

	*U*^11^	*U*^22^	*U*^33^	*U*^12^	*U*^13^	*U*^23^
Co1	0.0109 (3)	0.0182 (3)	0.0215 (3)	0.0007 (2)	0.0026 (2)	0.0016 (2)
S1	0.0148 (6)	0.0235 (7)	0.1153 (15)	0.0026 (5)	0.0068 (7)	0.0148 (8)
S2	0.0273 (6)	0.0273 (7)	0.0523 (9)	−0.0060 (5)	−0.0027 (6)	0.0136 (6)
O1	0.0404 (19)	0.0304 (18)	0.0242 (18)	−0.0125 (15)	0.0143 (15)	−0.0108 (14)
O2	0.0200 (15)	0.0262 (18)	0.0281 (19)	−0.0045 (14)	−0.0035 (14)	0.0116 (14)
N1	0.0224 (19)	0.0220 (19)	0.028 (2)	−0.0062 (16)	−0.0038 (16)	0.0029 (17)
N2	0.0141 (17)	0.026 (2)	0.018 (2)	0.0001 (15)	0.0031 (15)	−0.0023 (16)
N3	0.0214 (18)	0.0158 (18)	0.0170 (19)	−0.0014 (14)	0.0049 (15)	0.0010 (15)
N4	0.0164 (18)	0.027 (2)	0.039 (3)	0.0032 (17)	0.0019 (17)	0.0030 (18)
N5	0.0175 (18)	0.020 (2)	0.030 (2)	−0.0011 (16)	0.0022 (16)	0.0015 (17)
C1	0.043 (3)	0.027 (3)	0.033 (3)	−0.004 (2)	−0.016 (2)	0.000 (2)
C2	0.088 (5)	0.035 (3)	0.033 (3)	−0.003 (3)	−0.034 (3)	0.003 (3)
C3	0.108 (6)	0.065 (4)	0.018 (3)	0.005 (4)	0.001 (3)	−0.010 (3)
C4	0.065 (4)	0.053 (3)	0.017 (3)	−0.001 (3)	0.007 (3)	−0.007 (2)
C5	0.028 (2)	0.033 (3)	0.020 (3)	−0.009 (2)	0.003 (2)	−0.006 (2)
C6	0.017 (2)	0.035 (3)	0.028 (3)	−0.005 (2)	0.0004 (19)	−0.006 (2)
C7	0.021 (2)	0.071 (4)	0.045 (4)	0.004 (3)	0.011 (2)	−0.021 (3)
C8	0.018 (2)	0.026 (2)	0.030 (3)	0.0068 (18)	−0.0023 (19)	−0.003 (2)
C9	0.024 (2)	0.025 (2)	0.018 (2)	0.0018 (19)	−0.0060 (18)	0.0027 (19)
C10	0.027 (2)	0.022 (2)	0.020 (2)	−0.0041 (19)	−0.0013 (19)	0.0022 (19)
C11	0.035 (3)	0.028 (3)	0.022 (3)	−0.009 (2)	0.008 (2)	−0.002 (2)
C12	0.033 (3)	0.029 (3)	0.028 (3)	−0.011 (2)	0.017 (2)	−0.009 (2)
C13	0.035 (3)	0.026 (2)	0.016 (2)	−0.008 (2)	0.008 (2)	−0.0005 (19)
C14	0.016 (2)	0.025 (3)	0.049 (3)	−0.007 (2)	0.002 (2)	0.004 (2)
C15	0.0111 (19)	0.025 (2)	0.021 (2)	0.0056 (18)	−0.0041 (17)	−0.0041 (19)

### Geometric parameters (Å, °)

**Table:**

Co1—N4	2.057 (4)	C3—C4	1.372 (9)
Co1—N5	2.060 (4)	C3—H3	0.9500
Co1—N2	2.083 (3)	C4—C5	1.371 (7)
Co1—O2	2.137 (3)	C4—H4	0.9500
Co1—N1	2.171 (4)	C5—C6	1.486 (7)
Co1—N3	2.279 (4)	C6—C7	1.507 (6)
S1—C14	1.642 (5)	C7—H7A	0.9800
S2—C15	1.633 (5)	C7—H7B	0.9800
O1—C12	1.424 (5)	C7—H7C	0.9800
O1—C11	1.446 (5)	C8—C9	1.517 (6)
O2—H2A	0.83 (3)	C8—H8A	0.9900
O2—H2B	0.83 (3)	C8—H8B	0.9900
N1—C1	1.327 (6)	C9—H9A	0.9900
N1—C5	1.366 (6)	C9—H9B	0.9900
N2—C6	1.269 (6)	C10—C11	1.504 (6)
N2—C8	1.465 (6)	C10—H10A	0.9900
N3—C10	1.480 (5)	C10—H10B	0.9900
N3—C9	1.487 (5)	C11—H11A	0.9900
N3—C13	1.503 (5)	C11—H11B	0.9900
N4—C14	1.144 (6)	C12—C13	1.501 (6)
N5—C15	1.155 (5)	C12—H12A	0.9900
C1—C2	1.386 (8)	C12—H12B	0.9900
C1—H1	0.9500	C13—H13A	0.9900
C2—C3	1.385 (9)	C13—H13B	0.9900
C2—H2	0.9500		
			
N4—Co1—N5	93.44 (14)	N2—C6—C5	115.7 (4)
N4—Co1—N2	170.73 (15)	N2—C6—C7	125.4 (5)
N5—Co1—N2	91.43 (14)	C5—C6—C7	118.9 (4)
N4—Co1—O2	83.07 (14)	C6—C7—H7A	109.5
N5—Co1—O2	176.51 (13)	C6—C7—H7B	109.5
N2—Co1—O2	92.01 (13)	H7A—C7—H7B	109.5
N4—Co1—N1	95.53 (15)	C6—C7—H7C	109.5
N5—Co1—N1	95.59 (14)	H7A—C7—H7C	109.5
N2—Co1—N1	76.14 (14)	H7B—C7—H7C	109.5
O2—Co1—N1	84.63 (13)	N2—C8—C9	108.4 (3)
N4—Co1—N3	109.26 (15)	N2—C8—H8A	110.0
N5—Co1—N3	89.88 (13)	C9—C8—H8A	110.0
N2—Co1—N3	78.61 (13)	N2—C8—H8B	110.0
O2—Co1—N3	91.41 (12)	C9—C8—H8B	110.0
N1—Co1—N3	154.27 (13)	H8A—C8—H8B	108.4
C12—O1—C11	109.3 (3)	N3—C9—C8	111.9 (3)
Co1—O2—H2A	124 (4)	N3—C9—H9A	109.2
Co1—O2—H2B	126 (3)	C8—C9—H9A	109.2
H2A—O2—H2B	103 (5)	N3—C9—H9B	109.2
C1—N1—C5	118.9 (4)	C8—C9—H9B	109.2
C1—N1—Co1	127.7 (3)	H9A—C9—H9B	107.9
C5—N1—Co1	112.9 (3)	N3—C10—C11	112.0 (4)
C6—N2—C8	123.3 (4)	N3—C10—H10A	109.2
C6—N2—Co1	119.7 (3)	C11—C10—H10A	109.2
C8—N2—Co1	117.0 (3)	N3—C10—H10B	109.2
C10—N3—C9	109.6 (3)	C11—C10—H10B	109.2
C10—N3—C13	107.7 (3)	H10A—C10—H10B	107.9
C9—N3—C13	107.7 (3)	O1—C11—C10	110.4 (4)
C10—N3—Co1	116.0 (3)	O1—C11—H11A	109.6
C9—N3—Co1	101.6 (2)	C10—C11—H11A	109.6
C13—N3—Co1	113.9 (3)	O1—C11—H11B	109.6
C14—N4—Co1	158.4 (4)	C10—C11—H11B	109.6
C15—N5—Co1	166.6 (4)	H11A—C11—H11B	108.1
N1—C1—C2	122.1 (5)	O1—C12—C13	112.0 (4)
N1—C1—H1	119.0	O1—C12—H12A	109.2
C2—C1—H1	119.0	C13—C12—H12A	109.2
C3—C2—C1	119.1 (5)	O1—C12—H12B	109.2
C3—C2—H2	120.5	C13—C12—H12B	109.2
C1—C2—H2	120.5	H12A—C12—H12B	107.9
C4—C3—C2	118.8 (5)	C12—C13—N3	110.8 (3)
C4—C3—H3	120.6	C12—C13—H13A	109.5
C2—C3—H3	120.6	N3—C13—H13A	109.5
C5—C4—C3	120.0 (6)	C12—C13—H13B	109.5
C5—C4—H4	120.0	N3—C13—H13B	109.5
C3—C4—H4	120.0	H13A—C13—H13B	108.1
N1—C5—C4	121.2 (5)	N4—C14—S1	178.1 (5)
N1—C5—C6	115.3 (4)	N5—C15—S2	178.5 (4)
C4—C5—C6	123.5 (5)		

### Hydrogen-bond geometry (Å, °)

**Table:**

*D*—H···*A*	*D*—H	H···*A*	*D*···*A*	*D*—H···*A*
O2—H2A···O1^i^	0.83 (3)	1.88 (3)	2.701 (4)	172 (5)
O2—H2B···S1^ii^	0.83 (3)	2.36 (3)	3.161 (3)	162 (5)
C11—H11A···O2	0.99	2.40	3.121 (6)	130
C12—H12B···N4	0.99	2.62	3.511 (7)	150
C2—H2···S1^i^	0.95	2.85	3.774 (6)	165

Symmetry codes: (i) *x*, −*y*+1/2, *z*+1/2; (ii) *x*−1, *y*, *z*.
